# The Danish cancer pathway for patients with serious non-specific symptoms and signs of cancer–a cross-sectional study of patient characteristics and cancer probability

**DOI:** 10.1186/s12885-015-1424-5

**Published:** 2015-05-20

**Authors:** Mads Lind Ingeman, Morten Bondo Christensen, Flemming Bro, Søren T. Knudsen, Peter Vedsted

**Affiliations:** 1 Research Unit for General Practice, Aarhus University, Aarhus, Denmark; 2 Research Centre for Cancer Diagnosis in Primary Care (CaP), Aarhus University, Aarhus, Denmark; 3 Department of Public Health, Section for General Medical Practice, Aarhus University, Aarhus, Denmark; 4 Department of Endocrinology and Internal Medicine (MEA), Aarhus University Hospital, Noerrebrogade, Aarhus, Denmark

**Keywords:** Fast-track, Neoplasm, General practice, Diagnosis, Cancer symptoms, Denmark

## Abstract

**Background:**

A Danish cancer pathway has been implemented for patients with serious non-specific symptoms and signs of cancer (NSSC-CPP). The initiative is one of several to improve the long diagnostic interval and the poor survival of Danish cancer patients. However, little is known about the patients investigated under this pathway. We aim to describe the characteristics of patients referred from general practice to the NSSC-CPP and to estimate the cancer probability and distribution in this population.

**Methods:**

A cross-sectional study was performed, including all patients referred to the NSSC-CPP at the hospitals in Aarhus or Silkeborg in the Central Denmark Region between March 2012 and March 2013. Data were based on a questionnaire completed by the patient’s general practitioner (GP) combined with nationwide registers. Cancer probability was the percentage of new cancers per investigated patient. Associations between patient characteristics and cancer diagnosis were estimated with prevalence rate ratios (PRRs) from a generalised linear model.

**Results:**

The mean age of all 1278 included patients was 65.9 years, and 47.5 % were men. In total, 16.2 % of all patients had a cancer diagnosis after six months; the most common types were lung cancer (17.9 %), colorectal cancer (12.6 %), hematopoietic tissue cancer (10.1 %) and pancreatic cancer (9.2 %). All patients in combination had more than 80 different symptoms and 51 different clinical findings at referral. Most symptoms were non-specific and vague; weight loss and fatigue were present in more than half of all cases. The three most common clinical findings were ‘affected general condition’ (35.8 %), ‘GP’s gut feeling’ (22.5 %) and ‘findings from the abdomen’ (13.0 %). A strong association was found between GP-estimated cancer risk at referral and probability of cancer.

**Conclusions:**

In total, 16.2 % of the patients referred through the NSSC-CPP had cancer. They constituted a heterogeneous group with many different symptoms and clinical findings. The GP’s gut feeling was a common reason for referral which proved to be a strong predictor of cancer. The GP’s overall estimation of the patient’s risk of cancer at referral was associated with the probability of finding cancer.

**Electronic supplementary material:**

The online version of this article (doi:10.1186/s12885-015-1424-5) contains supplementary material, which is available to authorized users.

## Background

Cancer is the most common cause of death in Denmark and many other countries. One in five of all citizens in the developed world will die from cancer [1]. British and Danish cancer patients experience poorer cancer survival rates than patients from other western countries [2, 3]. Differences in public cancer awareness, health-care seeking behaviour, diagnostic pathways and treatment options have been suggested as important contributing factors [3]. Studies indicate that early diagnosis of cancer is important for improving the prognosis [4, 5]. The health care system must, therefore, provide medical services for prompt cancer diagnosis.

The majority of patients with cancer have a symptomatic presentation of the disease [6]. Symptoms are often diverse and may evolve over time as the cancer develops. In many health systems, general practitioners (GPs) form the first line of health care and provide medical advice to an unselected group of people. At the same time, GPs often act as ‘gatekeepers’ to ensure appropriate and timely flow of patients into the more specialized health services [7]. Thus, general practice plays a central role in diagnosing cancer [8–10]. Furthermore, the use of general practice has been shown to increase significantly several months before a patient is diagnosed with cancer [11]; this indicates an open ‘diagnostic window’.

To reduce the length of the diagnostic interval, several countries have implemented urgent referral cancer pathways [9, 12, 13] for patients with clinical suspicion of cancer [14]. In the UK, such pathway was introduced as the 2-week wait referral (2WW) system [15]. The first Danish Cancer Patient Pathways (CPPs) for diagnosis and treatment of suspected cancer were implemented in 2008; these are specific clinical pathways for several of the most common cancers/cancer sites [14, 16]. Once the GP refers the patient to a CPP, all diagnostic and treatment procedures will be promptly organised in well-defined processes; all relevant clinical investigations and treatments will be planned and booked within a given number of days. The aim of the CPP is to offer patients optimal diagnosis and treatment, which may ultimately improve their prognosis, and to provide better quality of life by reducing the insecurity that tends to accompany unwarranted delays.

Alarm symptoms of cancer and the related practice guidelines [17] are the primary focus of both the Danish and the British pathways [18, 19]. This approach may result in shorter diagnostic intervals [20] for patients with specific alarm symptoms. However, only approx. 40 % of all cancer patients seem to have benefitted from the implementation of the CPPs based on alarm symptoms as demonstrated by British and Danish studies [21, 22]. This is due to the fact that only half of cancer patients initially present symptoms classified as alarm symptoms by the GP [8, 21], findings from the UK indicate similar figures [20]. As a consequence of these findings, additional CPPs were implemented in Denmark in 2011 for patients with serious non-specific symptoms and signs of cancer (NSSC-CPP) [23]. These provided the Danish GPs with the opportunity to refer patients with serious non-specific symptoms for further diagnostic workup if cancer is suspected although no alarm symptoms (qualifying for specific CPP routes) are present [24]. However, the consequences of this urgent referral modality are not known at present. In particular, more information is needed on i) which patients are referred, ii) which factors constitute the basis of the referral and iii) whether or not the investigated patients have cancer.

This paper aims to describe the characteristics of patients referred from general practice to the Danish NSSC-CPP and to estimate the probability and distribution of cancers in this population.

## Methods

We performed a cross-sectional study including all patients aged 18 years or more who were referred to the NSSC-CPP at the hospitals in Aarhus or Silkeborg in the Central Denmark Region between 7 March 2012 and 27 March 2013. All identified patients were followed up for six months for the diagnosis of cancer.

### Setting and NSSC-CPP organisation

All Danish residents are entitled to tax-financed public health-care benefits with free access to health care. More than 98 % of Danish citizens are registered with a specific general practice. The GPs act as gatekeepers to the rest of the health-care system, except for emergencies [25]. During one year, 85 % of the Danish population is in contact with general practice.

**Fig. 1:**
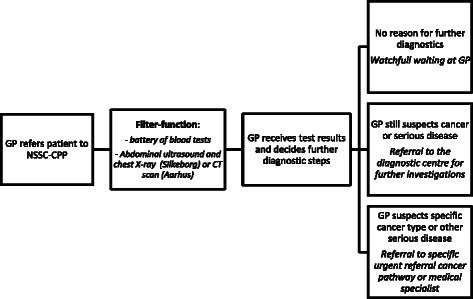
Organisation of the Danish NSSC-CPP

All patients referred from their GP to the NSSC-CPP underwent a filter function comprising three components: a battery of blood tests, a urine test and diagnostic imaging. The diagnostic imaging consisted of an abdominal ultrasound and a chest X-ray performed at Silkeborg hospital and a CT scan (with contrast) of chest, abdomen and pelvis performed at Aarhus University Hospital. The results of the diagnostic imaging were first assessed by a radiologist, and the GP subsequently interpreted all test results in combination and decided on further diagnostic steps to be taken. Such steps could be either watchful waiting or referral to a diagnostic centre for further investigations. If a specific disease or type of cancer was suspected, further steps could also involve referral to a medical specialist or another cancer-specific CPP (Fig. 1).

A diagnostic centre is a medical unit with comprehensive facilities for diagnostic investigation, including easy access to expertise in a wide range of relevant medical specialties (e.g. oncology, gynaecology, gastroenterological surgery, orthopaedics and radiology). NSSC-CPP patients referred to a diagnostic centre must undergo further investigations on the basis of presented symptoms and clinical findings (e.g. blood tests, diagnostic imaging, endoscopies and biopsies). Based on the findings, the patient is either referred to a CPP for a specific cancer, to a specific hospital department or back to the GP.

The Danish medical services are divided into five regions, and each of these regions must have at least one diagnostic centre. Approx. 15 centres have so far been established in Denmark.

### Identification of patients

All patients who underwent the filter function were identified and included. In the Silkeborg catchment area, eligible patients were identified by a digital marker on the battery of blood tests. At the hospital in Aarhus, all patients receiving CT scans as part of the filter function were identified with a particular code.

The unique civil registration number (CRN), which is assigned to all Danish citizens, links the medical records at the personal level across the Danish national registries [26]. Newly identified patients were extracted every two weeks, and we linked these data to the Health Service Registry (HSR) in the Central Denmark Region to identify the GP of each of the included patients.

Some referrals to the NSSC-CPP were made from hospital departments. To ensure inclusion of only relevant patients, we sent a letter to the GPs of the patients who were referred from the hospital to clarify whether the GP had been involved in the referral of this particular patient.

In total, 1899 referrals (1837 unique patients) were identified. We decided to consider two referrals of the same patient as two separate events if six or more months had passed between the referrals.

**Fig. 2:**
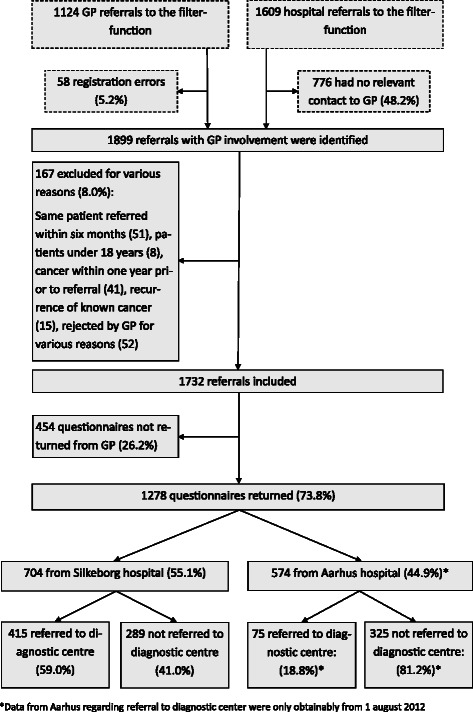
Referrals and patient inclusion for the NSSC-CPP

A total of 167 (8.0 %) referrals were excluded for the following reasons: same patient referred within six months (51 referrals), patient under 18 years (eight referrals), cancer within one year prior to current referral (41 referrals), recurrence of known cancer (15 referrals), questionnaire rejected and returned by the GP for various reasons, e.g. retirement of the referring GP (52 referrals). In total, 1732 referrals were included in the study (Fig. 2).

### Data collection

A pilot-tested paper questionnaire was sent to the GP of the identified patient no more than two weeks after inclusion of the patient in the study. This procedure was followed for all included patients. Non-respondents received a reminder after three weeks. In general practices with more than one GP, we asked the GP who was most familiar with the patient to complete the questionnaire. Participating GPs were remunerated for each completed questionnaire (DKK 121 corresponding to approx. EUR 16).

The GPs provided information regarding the patient’s symptoms, known chronic diseases and estimated risk of cancer at referral in addition to clinical findings, abnormal diagnostic test results and level of the GP’s ‘gut feeling’ (understood as clinical intuition) regarding possible serious disease. Furthermore, the date of the first symptom presentation to the GP/practice was reported.

Symptoms were defined as presence or absence of 21 specified symptoms at the time of referral, with the option to add other symptoms that were not listed. As far as possible, all symptoms were classified according to the International Classification of Primary Care, second edition (ICPC-2) [27]. Clinical findings were defined as the GP’s abnormal findings during the clinical examination of the patient. Diagnostic test results were defined as diagnostic tests that were considered abnormal and highly relevant for the overall pathological picture at the time of referral. In accordance with Stolper’s work, we define gut feeling as ‘a physician’s intuitive feeling that something is wrong with the patient, although there are no apparent clinical indications for this, or a physician’s intuitive feeling that the strategy used in relation to the patient is correct, although there is uncertainty about the diagnosis’ [28].

In line with the Aarhus Statement [13], the primary care interval was defined as the time from the patient’s first symptom presentation at the GP/practice until referral to the NSSC-CPP. To ensure accurate data, we used the registered inclusion date as the referral date, i.e. the electronically registered date at which the filter function had been ordered.

Data regarding each patient’s cancer diagnosis were retrieved from the Danish Cancer Registry (DCR) [29–31]. These data were available only for the period until 31 December 2012. Cancer diagnoses made after this date were retrieved from the National Patient Registry (NPR) until six months after the date for inclusion of the last patient. The identification of incident cancers from the NPR has proven to be reliable as 95 % of the cancer diagnoses are displayed after four months and with high validity [32]. The date of diagnosis in the NPR was defined as the first date of the hospital admission at which the cancer diagnosis was confirmed in the DCR. If the patient was diagnosed with ICD-10 codes C760–C800 (i.e. malignant neoplasm’s of ill-defined, other secondary and unspecified sites), we searched and replaced this code with a more cancer-specific diagnostic code if the diagnosis had been made no more than two months after the date at which the cancer incidence had first been registered.

Data collection regarding referral for further examination at the diagnostic centre at the hospital in Aarhus did not start until 1 August 2012. Thus, the data collection for the data shown in Table 4 started nearly five months later than the data collection from the hospital in Silkeborg.

### Statistical analyses

We used chi-square (χ2) test and Wilcoxon rank-sum test to identify differences between participating and non-participating GPs, to examine variations in the primary care interval between patients with and without cancer and to calculate the prevalence ratio (PR) in Table 5. The primary care intervals are presented as medians as well as 75 and 90 percentiles.

Cancer probability is presented as the percentage of included patients who were diagnosed with a new cancer within six months after the referral date. Associations between different patient characteristics and subsequent cancer diagnosis were estimated with prevalence rate ratios (PRRs) from a generalised linear model, both unadjusted and adjusted for age and gender, including 95 % confidence intervals (95 % CIs).

The statistical significance level was 0.05 or less. No alterations were made regarding missing data on presence or no presence of cancer. Stata statistical software v. 11 was used for the analyses.

### Ethics and approval

The study was approved by the Danish Data Protection Agency (j.no: 2011-41-6118) and the Danish Health and Medicines Authority (j.no: 7-604-04-2/301). This study needed no approval from the Danish National Committee on Health Research Ethics.

## Results

### Study population

A total of 1278 completed GP questionnaires (73.8 %) were returned and included in the analyses (Fig. 2). Five patients were included twice. No significant differences were found between referrals from participating GPs and non-participating GPs concerning hospital distribution, gender, age or probability of cancer diagnoses (Table 1).

**Table 1:** Characteristics of patients referred from participating GPs and from all included referrals

Variable		Referrals from participating GPs		All referrals including non-responders	
		*n* = 1278		*n* = 1732	
		n	%	N	%
**Hospital**					
	Silkeborg	705	55.2	927	53.5
	Aarhus	573	44.8	805	46.5
**Sex**					
	Female	671	52.5	821	52.6
	Male	607	47.5	911	47.7
**Age**					
	Mean	65.9 years		66.1	
	(Range, SD)	(18–98, 14.7)		(18–98, 14.7)	
**Age groups**					
	18-39 years	70	5.5	90	6.2
	40-54 years	179	14.0	234	16.1
	55-69 years	441	34.5	481	33.0
	70-79 years	345	27.0	368	25.3
	≥80 years	243	19.0	282	19.4
**Cancer:**					
	Yes	207	16.2	277	16.0
	No	1071	83.8	1455	84.0
Chronic diseases at referral*:					
	Hypertension	355	27.8	-	-
	Chronic lung disease	216	16.9	-	-
	Diabetes	153	12.0	-	-
	Ischaemic heart disease	142	11.1	-	-
	Chronic joint or rheumatic disease	134	10.5	-	-
	Light to medium mental disorder	125	9.8	-	-
	Osteoporosis	79	6.2	-	-
	Apoplexy	69	5.4	-	-
	Moderate to severe mental disorder	67	5.2	-	-

*Data based on returned questionnaires and therefore exclusively on participating GPs

### Patient characteristics

The mean age of patients included in the analyses was 65.9 years (sd: 14.7, range: 18–99), and 47.5 % were men. The most frequent chronic diseases at referral were hypertension, chronic lung disease and diabetes (Table 1).

A total of 82 different symptoms and 51 clinical findings were identified from the GP questionnaires (data not shown). The median number of symptoms was 3.0. Non-specific symptoms were the most predominant of all registered symptoms; weight loss and fatigue were both present in more than half of all referrals (Table 2). Symptoms associated with the highest probability of cancer were jaundice (42.9 %), dysphagia (36.7 %), neurological dysfunction (35.3 %) and lump/tumour (26.9 %) (Table 2).

**Table 2:** Symptoms, abnormal clinical findings and abnormal diagnostic test results among included patients at referral

	Total (*n* = 1269)	Patients with cancer n (%)
**Symptoms at referral**		
Weight loss	671 (52.5 %)	104 (15.5 %)
Fatigue	642 (50.2 %)	102 (15.9 %)
Pain	468 (36.6 %)	86 (18.4 %)
Nausea	352 (27.5 %)	65 (18.5 %)
Malaise	314 (24.7 %)	59 (18.8 %)
Vertigo	174 (13.6 %)	29 (16.7 %)
Change in bowel habits	137 (10.7 %)	24 (17.5 %)
Excessive sweating	128 (10.0 %)	15 (12.5 %)
Cough	114 (8.9 %)	15 (13.2 %)
Lump/tumour	108 (8.5 %)	29 (26.9 %)
No symptom	33 (2.6 %)	7 (21.2 %)
**Abnormal clinical findings at referral**		
Affected general condition	457 (35.8 %)	80 (17.5 %)
GP’s ‘gut feeling’	287 (22.5 %)	69 (24.0 %)
Abdomen	166 (13.0 %)	35 (21.1 %)
Skin	61 (4.8 %)	12 (19.7 %)
Extremity	56 (4.4 %)	10 (17.9 %)
Lungs	51 (4.0 %)	7 (13.7 %)
Lymph node	44 (3.4 %)	12 (27.3 %)
Weight loss	35 (2.7 %)	3 (8.8 %)
Joints	31 (2.4 %)	3 (9.7 %)
Neurological dysfunction	30 (2.4 %)	8 (26.7 %)
**Abnormal diagnostic test results at referral**		
Blood sample at GP	619 (48.4 %)	104 (16.8 %)
Blood sample at hospital	253 (19.8 %)	37 (14.6 %)
Diagnostic imaging	192 (15.0 %)	32 (16.7 %)
Urine sample	2 (0.2 %)	1 (50.0 %)

The three most common clinical findings were affected general condition (35.8 %), the GPs’ gut feeling (22.5 %) and abdominal findings (13.0 %). The highest probability of cancer was found for enlarged lymph nodes (27.3 %), neurological findings (26.7 %), the GPs’ gut feeling (24.0 %) and abdominal findings (21.1 %) (Table 2).

Abnormal diagnostic test results were primarily related to blood samples and diagnostic imaging, and no single diagnostic test result was associated with a particularly high probability of cancer.

### Cancer and primary care interval

After six months, 16.2 % of all patients had a cancer diagnosis. The most common cancer types were lung cancer (17.9 %), colorectal cancer (12.6 %), hematopoietic tissue cancer (10.1 %) and pancreatic cancer (9.2 %) (Table 3). In comparison, the most common cancer types in Denmark in general for men are prostate cancer, lung cancer, colon cancer and urinary tract cancer, while the most common types for women are breast cancer, lung cancer, colon cancer and malignant melanoma.

**Table 3:** Diagnosed cancers among patients with serious non-specific cancer symptoms referred from participating GP; primary care interval shown as median, 75 % and 90 % percentiles

Cancer type	Numbers (% of all cancers)	Median (days)	75 percentile	90 percentile
All cancer patients	207 (100 %)	15	72	130
Lung cancer	37 (17.9 %)	19.5	77.5	127
Colorectal cancer	26 (12.6 %)	11	56	110
Hematopoietic tissue cancer	21 (10.1 %)	19	85	278
Pancreatic cancer	19 (9.2 %)	7	22	51
Oesophagus, stomach and small intestine cancer	17 (8.2 %)	32.5	88	130
Breast cancer	13 (6.3 %)	8	24	35
Liver and biliary system cancer	11 (5.3 %)	7	49	80
Kidney cancer	11 (5.3 %)	35	69	168
Metastasis	11 (5.3 %)	51	100	345
Prostate cancer	10 (4.8 %)	53	131.5	357
Brain cancer	5 (2.4 %)	21	21	52
Cervix, ovarian and uterus cancer	4 (1.9 %)	29	69.5	96
Malignant melanoma	4 (1.9 %)	12.5	79	135
Soft tissue cancer	4 (1.9 %)	36.5	79	99
Unspecified cancer	4 (1.9 %)	123	365	365
Lip, oral and pharynx cancer	2 (1.0 %)	9	9	9
Thyroid cancer	2 (1.0 %)	6	8	8
Other cancers*	6 (2.9 %)	34	74	108

*Ill-defined digestive organ cancer: larynx cancer, chest cavity cancer, sternum cancer and clavicle cancer, penis cancer and testicle cancer

The median primary care interval for patients diagnosed with cancer was 15 days; the 75 and 90 percentiles were 72 days and 130 days, respectively. Breast, liver and biliary cancer patients seemed to have shorter than average primary care intervals, while patients with metastases or cancer of the prostate, hematopoietic tissue, oesophagus, stomach or small intestine seemed to have longer primary care intervals than all other patients (Table 3). However, the study population was too small to provide any statistical precision for these estimates.

Men generally had a significantly higher probability of cancer than women when referred (adjusted PRR = 1.32 (95 % CI: 1.03-1.70)) (Table 4).

**Table 4:** Distribution of referrals, cancer probability, crude PRR and adjusted PRR according to referral characteristics, primary care interval, GP’s suspicion of cancer and GP’s gut feeling

			Referrals (%)	Probability of cancer (%)	Crude PRR for cancer (95% CI)	Adjusted PRR for cancer (95% CI)^a^
All			1278 (100%)	207 (16.2%)		
Hospital	Silkeborg		705 (55.2%)	101 (14.3%)	1 (ref)	1 (ref)
	Aarhus		573 (44.8%)	106 (18.5%)	1.29 (1.01–1.66)	1.22 (0.95–1.56)
Referral to further examination at diagnostic centre	Silkeborg	Yes	415 (59.0%)	49 (11.8%)	1 (ref)	1 (ref)
		No	289 (41.0%)	52 (18.0%)	1.64 (1.05-2.50)	1.62 (1.05-2.50)
	Aarhus	Yes	75 (18.8%)	12 (16.0%)	1 (ref)	1 (ref)
		No	325 (81.2%)	63 (19.4%)	1.26 (0.64-2.48)	1.22 (0.62-2.41)
Sex	Female		671 (52.5%)	95 (14.2%)	1 (ref)	1 (ref)
	Male		607 (47.5%)	112 (18.5%)	1.30 (1.02-1.67)	1.32 (1.03-1.70)
Age group	18-39 years		70 (5.5%)	3 (4.3%)	0.96 (0.26-3.51)	0.95 (0.26-3.49)
	40-54 years		179 (14.0%)	8 (4.5%)	1 (ref)	1 (ref)
	55-69 years		441 (34.5%)	80 (18.1%)	4.06 (2.00-8.22)	4.01 (1.98-8.12)
	70-79 years		345 (27.0%)	73 (21.2%)	4.73 (2.33-9.60)	4.76 (2.35-9.64)
	≥ 80 years		243 (19.0%)	43 (17.7%)	3.96 (1.91-8.21)	3.31 (1.90-8.15)
Patients with previous cancer	No		1134 (88.7%)	186 (16.4%)	1 (ref)	1 (ref)
	Yes		144 (11.3%)	21 (14.6%)	0.89 (0.59-1.35)	0.80 (0.52-2.20)
Symptoms at referral	0		23 (1.8%)	6 (26.1%)	1.96 (0.92-4.15)	1.88 (0.89-3.95)
(n=1254)	1		240 (19.0%)	32 (13.3%)	1 (ref)	1 (ref)
	2		276 (21.9%)	31 (11.2%)	0.84 (0.53-1.34)	0.82 (0.52-1.29)
	3		278 (22.0%)	47 (16.9%)	1.27 (0.84-1.92)	1.26 (0.84-1.91)
	4		206 (16.3%)	38 (18.5%)	1.38 (0.90-2.13)	1.38 (0.90-2.12)
	5		118 (9.3%)	27 (22.9%)	1.72 (1.08-2.72)	1.68 (1.06-2.65)
	≥6		122 (9.7%)	28 (18.9%)	1.41 (0.87-2.30)	1.35 (0.83-2.18)
Clinical findings at referral	0		147 (3.3%)	9 (6.1%)	1 (ref)	1 (ref)
	1		580 (52.4%)	80 (13.8%)	2.25 (1.16-4.82)	1.98 (1.02-3.84)
(n=1100)	2		297 (26.9%)	67 (22.6%)	3.68 (1.89-7.18)	3.04 (1.56-5.92)
	≥3		82 (7.4%)	19 (23.2%)	3.78 (1.80-7.98)	3.25 (1.55-6.81)
Diagnostic test results at referral	0		187 (17.1%)	13 (7.0%)	1 (ref)	1 (ref)
	1		565 (51.7%)	98 (17.4%)	2.50 (1.34-4.34)	2.16 (1.24-3.77)
(n=1086)	2		267 (24.4%)	41 (16.4%)	2.21 (1.22-4.01)	1.95 (1.07-3.53)
	≥3		75 (6.9%)	13 (17.3%)	2.49 (1.21-5.13)	2.28 (1.11-4.66)
Number of chronic diseases at referral	0		295 (24.5%)	50 (17.0%)	1 (ref)	1 (ref)
	1		403 (33.5%)	62 (15.4%)	0.91 (0.65-1-28)	0.73 (0.52-1.02)
(n=1199)	2		286 (23.5%)	48 (16.8%)	0.99 (0.69-1.42)	0.71 (0.49-1.02)
	≥3		220 (18.2%)	34 (15.5%)	0.91 (0.61-1.36)	0.63 (0.42-0.95)
Primary care interval^b^	<1 month		723 (56.6%)	117 (16.2%)	1 (ref)	1 (ref)
(n=1131)	1-2 months		156 (12.2%)	20 (12.8%)	0.79 (0.51-1.23)	0.81 (0.52-1.26)
	2-3 months		79 (6.2%)	16 (20.3%)	1.25 (0.78-2.00)	1.31 (0.82-2.07)
	3-4 months		52 (4.1%)	12 (23.1%)	1.43 (0.85-2.41)	1.42 (0.85-2.39)
	4-5 months		29 (2.3%)	6 (20.7%)	1.28 (0.62-2.66)	1.36 (0.67-2.76)
	5-6 months		17 (1.3%)	3 (17.7%)	1.10 (0.39-3.09)	1.26 (0.47-3.39)
	>6 months		222 (17.3%)	33 (14.9%)	0.92 (0.64-1.31)	0.90 (0.64-1.29)
GP’s estimation of patient’s risk of cancer at referral	0-20%		448 (36.8%)	36 (8.0%)	1 (ref)	1 (ref)
	21-40%		195 (16.0%)	24 (12.3%)	1.53 (0.94-2.50)	1.43 (0.88-2.33)
	41-60%		314 (25.8%)	47 (15.0%)	1.86 (1.24-2.81)	1.69 (1.12-2.56)
(n=1208)	61-80%		155 (12.6%)	41 (26.5%)	3.29 (2.19-4.95)	2.96 (1.96-4.48)
	81-100%		104 (8.6%)	52 (50.0%)	6.22 (4.31-8.99)	5.30 (3.62-7.76)
Did gut feeling influence the decision of referral?	No		287 (24.6%)	46 (16.0%)	1 (ref)	1 (ref)
	A little		224 (19.2%)	25 (11.2%)	0.66 (0.39-1.11)	0.65 (0.38-1.10)
	Some		425 (36.4%)	63 (14.8%)	0.91 (0.60-1.38)	0.86 (0.56-1.31)
(n=1168)	Much		182 (15.6%)	43 (23.6%)	1.62 (1.02-2.58)	1.55 (0.97-2.48)
	Very much		50 (4.3%)	17 (34.0%)	2.70 (1.39-5.25)	2.57 (1.31-5.05)

^a^Adjusted for age and gender

GP: General Practitioner

^b^Medians are used to categorise the groups

PRR: Prevalence Rate Ratio

A more detailed overview of symptoms and clinical findings found to be highly predictive of cancer is presented in Additional file 1.

### Cancer probability in different referral groups

Referred patients with five symptoms had a significantly higher probability of having cancer than patients referred with only one symptom (adjusted PRR = 1.68 (95 % CI: 1.06-2.65)) (Table 4). The presence of one or more clinical and/or diagnostic test results implied a significantly higher probability of finding cancer (Table 4).

Patients from Aarhus constituted 44.8 % of the referrals. These patients had a significantly higher probability of cancer than the patients referred to the hospital in Silkeborg (although not in the adjusted analysis) (Table 4).

In total, 59.0 % of the patients from Silkeborg were referred to further examination at the diagnostic centre compared to 18.8 % of the patients from Aarhus. A higher probability of cancer was found among patients who had not been referred to further examination compared to patients who had been referred. However, this difference was only statistically significant in the group of patients from Silkeborg (Silkeborg: adjusted PRR = 1.62 (95 % CI: 1.05-2.50); Aarhus: adjusted PRR = 1.22 (95 % CI: 0.62-2.41)).

The number of chronic diseases and the length of the primary care interval showed no significant associations with the probability of cancer (Table 4).

A strong association was found between the GP’s assessments of estimated cancer risk at referral and the probability of finding cancer (Table 4).

The GPs’ estimations were generally higher than the actual probability of cancer. The probability of cancer was higher if the GP had reported ‘strong’ or ‘very strong’ compared to ‘no’ gut feeling. Furthermore, GP gut feeling showed an association with the four most common clinical findings (weight loss, fatigue, affected general condition and abnormal blood sample) for patients diagnosed with cancer (Prevalence ratio: 1.50 (95 % CI: 0.82-2.75)) (Table 5).

**Table 5:** Association between GP gut feeling and the four most common findings in cancer patients

		Four most common findings*		
		At least one	None	Total
GPs’ gut feeling	Yes	60	9	169
	No	109	29	138
	Total	169	38	207

Prevalence ratio: 1.50 (95 % CI: 0.82-2.75)

*Weight loss and fatigue (two most common symptoms), affected general condition (most common clinical finding) and abnormal blood sample at GP (most common abnormal diagnostic test result)

## Discussion

### Main findings

NSSC-CPP referred patients were a heterogeneous group with over 80 different symptoms, 51 different clinical findings and wide variations in number of symptoms per referral. The most frequent symptoms were non-specific and vague symptoms, which are also very frequent reasons for consultations in general practice [33]. The term ‘non-specific symptom’ is used as opposed to specific alarm symptoms as non-specific symptoms are not necessarily indicative of a specific cancer type, but may suggest several cancers or other diseases. Only a few symptoms were highly predictive of cancer; most of these were rare (<2 % of patients), except for lump/tumour which was present in almost 9 % of the patients. The GP’s estimation of the patient’s risk of cancer at referral showed an expected correlation with the actual probability of cancer. However, it should be noted that the GP’s estimated risk was almost twice the size of the actual probability of cancer.

The overall probability of cancer was 16 %. Cancer was found more often in men than in women, which might be explained by the fact that breast cancer often presents with an alarm symptom [34]. In addition, referred men tended to have a higher probability of cancer than referred women [35, 36].

Affected general condition was the most common clinical finding and the GP’s gut feeling was another important clinical finding, which also showed a high probability of cancer (24.0 %). As seen in Table 4, little influence of gut feeling was less predictive of cancer than no influence, which may be because some patients have clear symptoms where gut feeling has minor importance. Nonetheless, an association was found between the most common findings and gut feeling, as shown in Table 5. These findings indicate that more research is needed to further explore the role of gut feeling in early diagnosis of serious disease. Our study did not allow identification of the specific components of this gut feeling, but it seems to embrace several clinical aspects that in combination increase the patient’s probability of cancer.

The primary care interval for all cancer patients diagnosed in this study was markedly longer than the interval found in previous studies [37, 38]. The long primary care trajectory before referral underlines the complexity of diagnosing these patients, but also stresses the need for quick and easy access to diagnostic investigations [39], including earlier referral by the GP despite non-specific symptoms.

The higher probability of cancer among patients not referred to further examination at a diagnostic centre may be explained by the separation of patients with specific cancer findings through the filter function; these patients are referred to specific CPPs or other pathways and not to the diagnostic centre. This indicates that the filter function prior to the referral to the diagnostic centre is useful. However, some patients who were terminated by the GP without further examination (watchful waiting) may actually have had a cancer or another serious disease. The present study did not gain insight into this issue, and further research in this area is needed.

The lower percentage (18.8 %) of referrals from the hospital in Aarhus to further examination at the diagnostic centre might partly be explained by the use of an initial CT scan, which may be more effective as a diagnostic instrument and thus may reduce the need for referral to further diagnostic workup. However, it could also be false assurance as no difference was found in the proportions of cancer between non-referred patients and patients referred to the diagnostic centre in Aarhus. Furthermore, the NSSC-CPP at the hospital in Silkeborg had been implemented several years before the NSSC-CPP in Aarhus. This difference may also have affected the number of GPs who chose to refer to the diagnostic centre.

### Strengths and weaknesses of the study

A major strength of this study is the prospective design, which allowed us to include all patients referred to the NSSC-CPP and not only already diagnosed cancer patients. Although we included patients prospectively, the questionnaires were sent out retrospectively, and this may have introduced recall bias. To minimise recall bias, we posted our questionnaire to the GP no more than two weeks after inclusion of the patient, and the diagnostic workup for many patients had not been finished by the time the GP received the questionnaire. This also minimized possible information bias as the GPs did not know the results of the referral for many of the patients. To further minimize recall bias, we encouraged the GPs to consult their electronic medical records when filling in the questionnaire. Nevertheless, recall bias might be more pronounced for patients referred through a hospital department as the GPs referred the patients to a hospital department before the patients were referred to the NSSC-CPP by the hospital. Further data on this potential recall bias were not available. Lack of complete information in some questionnaires might have introduced information bias, but this is unlikely to have influenced the estimated probability of cancer or the reported clinical findings.

The register data are considered precise and valid as the cancer information in the DCR was registered prospectively. The DCR has an almost complete registration of all Danish cancer data and has been shown to be accurate [29]. We used the NPR to identify cancer patients diagnosed in 2013, and this method of identifying cancer patients has been reported to have an accuracy of 95 % after four months [32]. The introduced misclassification is considered to be non-differential.

The GP response rate is comparable to similar studies using GP questionnaires [34, 37] and must be considered high, which limits potential selection bias. Still, non-responding GPs may have had patients with special characteristics although a non-response analysis revealed no differences between patients of participating GPs and patients of non-participating GPs.

Although ’gut feeling’ is a well-known and common phenomenon among GPs [28], this notion may have introduced a problem regarding the construct validity as it is uncertain whether GPs regard ‘gut feeling’ in the same way. Furthermore, ‘gut feeling’ can be difficult to separate from e.g. the GP’s estimation of the patient’s risk of cancer in this study design. The association between gut feeling and the four most common findings indicates that gut feeling is often seen in combination with other findings. Further sub analysis showed that no symptoms, clinical findings or abnormal diagnostic test results were stated in the medical records for only 11 of the patients; none of these patients were registered with a GP gut feeling. Furthermore, the fact that the probability of cancer appeared higher with no gut feeling (compared to little gut feeling) indicates that presence of clear signs of cancer does not generally prompt activation of gut feeling. Our results warrant further studies into the importance of ‘gut feeling’ in early detection of cancer.

### Comparison with other studies

Bosch et al. [40] published a paper on referrals from GPs to a quick diagnostic unit (QDU) similar to the one described in this paper, but their aim was different from ours. The study showed that 30 % of the patients referred directly to the QDU had cancer compared to the 16 % found in our study. Data from the UK have shown that 11 % of the patients referred to the ordinary urgent referral pathways were diagnosed with cancer [22]. Apart from the study by Bosch et al. [40], we are unaware of any published studies examining and quantifying GP referrals to NSSC-CPPs and related outcomes.

An earlier study confirmed that action should be taken when the GP suspects serious disease as these patients have a high risk of a new diagnosis of cancer or another serious disease within 2 months [41]. Furthermore, Hamilton has also highlighted the importance of the GP’s suspicion [6]. Our study adds to this evidence within primary care diagnostics.

Jensen et al. [21] documented that only 40 % of the Danish cancer patients were referred to a ‘cancer specific’ CPP. This finding stresses the importance of providing the GPs with diagnostic tools like the NSSC-CPP as well as direct access to diagnostic investigations [39, 42, 43].

## Conclusions

This study documents that 16.2 % of all patients referred through the Danish NSSC-CPP because of non-specific serious symptoms had cancer. Patients referred to the NSSC-CPP were a heterogeneous group with many different symptoms and clinical findings. The GP’s gut feeling was a common clinical finding which was a strong predictor of cancer. Likewise, the GP’s assessment of the patient’s risk of cancer at referral was also strongly associated with the actual probability of finding cancer.

## Additional file

Below is the link to the electronic supplementary material.

Additional file 1: **Symptoms and abnormal clinical findings highly predictive of cancer.**
