# Supplementary material for: The Danish cancer pathway for patients with serious non-specific symptoms and signs of cancer–a cross-sectional study of patient characteristics and cancer probability
Source: BMC Cancer. 2015 May 20;15:421. doi: 10.1186/s12885-015-1424-5 (PMC4445271; doi:10.1186/s12885-015-1424-5)
Supplement: Supplementary file 1 — Symptoms and abnormal clinical findings highly predictive of cancer. [file 12885_2015_1424_MOESM1_ESM.pdf]

**Appendix 1.** Symptoms and abnormal clinical findings highly predictive of cancer

| | Total (n=1269) | Patients with cancer n (%) |
|---|---|---|
| **Symptoms at referral** | | |
| Feeling of hunger | 3 (0.2%) | 2 (66.7%) |
| Paralysis/limb weakness | 4 (0.3%) | 2 (50.0%) |
| Neurological symptom | 18 (1.4%) | 8 (44.4%) |
| Jaundice | 14 (1.1%) | 6 (42.9%) |
| Abdominal distension | 22 (1.7%) | 9 (40.9%) |
| Dysphagia | 30 (2.4%) | 11 (36.7%) |
| Back pain | 9 (0.7%) | 3 (33.3%) |
| Tingling fingers/feet/toes | 6 (0.5%) | 2 (33.3%) |
| Joint symptom | 7 (0.6%) | 2 (28.6%) |
| Memory loss | 7 (0.6%) | 2 (28.6%) |
| Blood in stool | 35 (2.8%) | 9 (27.7%) |
| Lump/tumor | 107 (8.4%) | 29 (27.1%) |
| **Clinical findings at referral** | | |
| Bone soreness | 3 (0.2%) | 2 (66.7%) |
| Cognitive problems | 3 (0.2%) | 2 (66.7%) |
| Icterus | 10 (0.8%) | 5 (50.0%) |
| Ascites | 2 (0.2%) | 1 (50.0%) |
| Abnormal finding in mamma | 6 (0.5%) | 2 (33.3%) |
| Back pain | 6 (0.5%) | 2 (33.3%) |
| Anaemia | 3 (0.2%) | 1 (33.3%) |
| Level of conscience | 13 (1.0%) | 4 (30.8%) |
| Neurological finding | 29 (2.3%) | 8 (27.6%) |
| Abnormal lymph glands | 43 (3.4%) | 11 (25.6%) |
| GP's gut feeling | 287 (22.6%) | 73 (25.4%) |
| Exploratio rectalis | 8 (0.6%) | 2 (25.0%) |
